# The Changes of Lipidomic Profiles Reveal Therapeutic Effects of Exenatide in Patients With Type 2 Diabetes

**DOI:** 10.3389/fendo.2022.677202

**Published:** 2022-03-31

**Authors:** Lin Zhang, Yanjin Hu, Yu An, Qiu Wang, Jia Liu, Guang Wang

**Affiliations:** Department of Endocrinology, Beijing Chaoyang Hospital, Capital Medical University, Beijing, China

**Keywords:** exenatide, lipidomics, serum, type 2 diabetes, treatment

## Abstract

**Objective:**

Exenatide has been demonstrated beneficial effects on patients with type 2 diabetes mellitus (T2DM) regarding lipid metabolism. However, the potential mechanism remains unclear. We used a lipidomic approach to evaluate lipid changes in response to treatment with exenatide in T2DM patients.

**Methods:**

Serum lipidomic profiles of 35 newly diagnosed T2DM patients (before and after exenatide treatment) and 20 age-matched healthy controls were analyzed by ultrahigh-performance liquid chromatography-tandem quadrupole time-of-flight mass spectrometry.

**Results:**

A total of 45 lipid species including sphingomyelins (SMs), ceramides (CERs), lysophosphatidylcholines (LPCs), phosphatidylethanolamines (PEs), lysophosphatidylethanolamines (LPEs) and phosphatidylcholines (PCs) were identified in all participants. Compared to the healthy controls, 13 lipid species [SM (d18:1/18:0, d18:1/18:1), Cer (d18:1/18:0, d18:1/16:0, d18:1/20:0, d18:1/24:1), LPC (15:0, 16:0, 17:0), PC (19:0/19:0), LPE (18:0) and PE (16:0/22:6, 18:0/22:6)] were markedly increased in the T2DM group, while PE (17:0/17:0) and PC (18:1/18:0) were decreased (*P* < 0.05). The serum SM (d18:1/18:0, d18:1/18:1), LPC (16:0), and LPE (18:0) were significantly decreased after exenatide treatment, which was accompanied by the amelioration of lipids and glycemic parameters (TC, LDL-C, ApoA-I, FCP and HbA_1c_) in T2DM patients. The chord diagrams showed distinct correlation patterns between lipid classes and subclasses among healthy controls, T2DM patients before and after exenatide treatment.

**Conclusion:**

Our results revealed that the therapeutic benefits of exenatide on T2DM might be involved in the improved lipid metabolism, especially SM, LPC, and LPE.

**Clinical Trial Registration:**

ClinicalTrials.gov, identifier NCT03297879.

## Introduction

Type 2 diabetes mellitus (T2DM), frequently associated with a cluster of endocrine disturbances including obesity, dysglycemia, and dyslipidemia, has increasingly become a global public health problem with wide concern owing to its high incidence and a series of adverse complications (1–3). Dyslipidemia in T2DM demonstrated an increased risk for microvascular and macrovascular complications, which is the reflection of lipid metabolism disorders (4, 5). Dysregulation of lipid metabolism may be related to fatty acid regulation, β-oxidation, mitochondrial dysfunction, endoplasmic reticulum stress, and inflammation in diabetes complications (6, 7). Excess uptake of free fatty acids from food flows into the synthesis of phospholipids, glycerolipids, and sphingolipids, which may reflect the main driving force behind the lipotoxicity leading to T2DM (8). Hence, a better understanding of the alterations in the lipid molecules may help to uncover the mechanistic links in T2DM. Lipidomics has emerged as an important branch of metabolomics for identifying and quantifying the cell lipids in response to pathophysiological stimuli (9). Currently, lipidomics has been applied in T2DM for identifying possible predictive and diagnostic biomarkers (10–12). Meanwhile, a previous study, using metabolomic and lipidomic profiling methods, found that arabinoxylan alleviated hypercholesterolemia and hyperlipidemia in type 2 diabetic rats by regulating bile acid and lipid metabolites (13). This finding suggests lipidomics has also been used to assess the alteration in circulating lipid composition to define the therapeutic effects of T2DM.

Exenatide, a glucagon-like peptide-1 (GLP-1) analog, has been commonly used for treating T2DM (14). Our previous studies have found that exenatide can not only reduce body weight, improve glucose metabolism and insulin resistance but also improve lipid metabolism (15, 16). Tang L et al. identified several metabolites as potential markers for the therapeutic efficacy of exenatide in PCOS by plasma metabolomic profiles, which provide evidence for further clinical application (17). However, exenatide on lipidomic abnormalities and its associated effective lipid molecular biomarkers have never been studied in T2DM patients. Therefore, our study aimed to evaluate the serum lipidomic profiles in healthy subjects and T2DM patients before and after exenatide administration, using an ultrahigh-performance liquid chromatography-tandem quadrupole time-of-flight mass spectrometry (UPLC-QTOF-MS) approach. For completeness, we also evaluated the effects of exenatide therapy on T2DM-related metabolic indicators and their relationship with lipidomic changes.

## Materials and Methods

### Participants

Thirty-five newly diagnosed T2DM patients and twenty age-matched healthy controls were successively enrolled from the department of endocrinology of the Beijing Chao-yang Hospital Affiliated with Capital Medical University during September 2013 and October 2015. The diagnosis of T2DM was according to the American Diabetes Association standard (18). All patients fulfilled the following inclusion criteria: (i) no use of antidiabetic drugs; (ii) aged 20 to 65 years; (iii) body mass index (BMI) ≥ 24 kg/m^2^ (19); (iv) Hemoglobin A1c (HbA_1c_) ≥ 7%. The exclusion criteria of all participants were: type 1 diabetes, pregnancy, pancreatitis, coronary artery disease, hepatic impairment, renal dysfunction, intestinal surgery, hematological disorders, infectious disease and cancer.

### Study Design

All subjects received a screening assessment at baseline and T2DM patients were also evaluated clinically after the 12-week study. Blood samples were collected after overnight fasting and stored at −80°C until lipidomics analysis. The general methodology for clinical measurements is described in our previous study (16, 20). Eligible T2DM subjects received a continual subcutaneous injection of exenatide for a total of 12 weeks (5 µg twice a day for 4 weeks, followed by 10 µg twice a day for 8 weeks). Lifestyle interventions (diet and exercise) were delivered to all patients by an experienced nurse according to the Guidelines for Prevention and Treatment of Type 2 Diabetes in China (2013 Edition). The study adhered to the Declaration of Helsinki. The study protocol was approved by the Ethics Committee of Beijing Chao-yang Hospital Affiliated with Capital Medical University. All participants gave their written informed consent.

### Lipidomic Analysis

Serum lipids were determined by the UPLC-QTOF-MS approach according to the previous study (21–23). Samples were extracted by chloroform-methanol solution and analyzed in an LC-20AXR Rapid Separation LC system (Shimadzu, Kyoto, Japan). Quantification of all lipids was performed by negative mode electrospray ionization mass spectrometry using AB Triple quadrupole time-of-flight 5,600 mass spectrometer (AB SCIEX, Foster City, CA, USA). To ensure quantitative accuracy, quality control (QC) samples were included every 15 samples, features with the coefficient of variation (CV) over 15% were eliminated. Additional details were provided in **Supplementary Methods**.

### Statistical Analysis

The data are expressed as the mean ± SD or as the median (quartile). Differences of baseline clinical and biochemical measurements between patients with T2DM and healthy controls were evaluated using the independent sample t-test for normally distributed variables or the Mann-Whitney test for skewed distributed variables. Pearson chi-squared test was used for categorical variables. Within-group comparisons before and after exenatide were performed using paired-sample t-tests or paired-sample Wilcoxon test to evaluate the changes from baseline. Pearson or Spearman correlation coefficients were calculated between serum lipid species and clinical parameters. Statistical analyses were performed with SPSS 23.0 (IBM Corporation, NY, USA). Data visualization was achieved by heatmaps and chord diagrams, both of which were plotted using R (version 3.5.1). The chord diagrams were constructed using significantly correlated lipids in each lipid class of different groups. Lipid pairs with a correlation P-value < 0.05 were considered as significant correlations.

## Results

### Baseline Clinical Characteristics of All Participants

Baseline characteristics of the T2DM and control groups are summarized in **Table 1**. The T2DM group had significantly higher BMI, FBG, HbA_1c_, and HOMA-IR, and lower HDL-C, ApoA-I, and HOMA-β compared with the control group (HDL-C and HOMA-IR: *P* < 0.05; BMI, FBG, HbA_1c_, ApoA-I, and HOMA-β: *P* < 0.01, **Table 1**). There were no significant differences in TC, LDL-C, TG, ApoB, FINS, and FCP between the two groups.

**Table 1:** Baseline characteristics of study participants.

	Matched control group (n =20)	Type 2 diabetes group (n = 35)	
		Baseline	Change after exenatide treatment
Age, years	46.06 ± 14.04	43.85 ± 12.05	
Sex, M/F	10/10	23/12	
BMI, kg/m^2^	26.36 ± 3.55	30.92 ± 5.04**	-1.88 (-3.46 to -0.33)^##^
TC, mmol/L	4.91 (4.28-5.47)	4.68 (4.03 to 5.54)	-0.46 (-1.11 to 0.7)^##^
LDL-C, mmol/L	2.83 (2.53-3.24)	2.82 (2.44 to 3.55)	-0.33 (-0.71 to 0.19)^#^
HDL-C, mmol/L	1.25 (1.09-1.74)	1.11 (0.95 to 1.31)*	0.06 (-0.11 to 0.19)
TG, mmol/L	1.32 (1.04-1.83)	1.81 (1.22 to 3.00)	-0.51 (-1.05 to 0.22)^##^
ApoA-I, g/L	1.59 (1.37-1.77)	1.11 (0.95 to 1.27)**	0.04 ± 0.26
ApoB, g/L	0.87 ± 0.18	0.91 ± 0.18	-0.09 ± 0.21^#^
FBG, mmol/L	5.1 (4.58-5.72)	8.28 (6.80 to 11.92)**	-1.52 (-4.61 to -0.11)^##^
FINS, μIU/mL	9.88 (5.41-13.5)	10.04 (6.04 to 14.2)	1.15 (-4.42 to 3.41)
FCP, ng/mL	2.81 ± 0.94	2.88 ± 1.02	-0.03 ± 0.81
HbA_1c_, %	5.5 (5.3-6.3)	10.1 (7.65 to 11.23)**	-3.14 ± 2.04^##^
HOMA-IR	2.23 (1.22-3.04)	3.77 (2.26 to 6.10)*	-1.48 ± 2.74^##^
HOMA-β	129.64 (52.82-240.15)	39.73 (22.75 to 66.1)**	24.71 (-0.59 to 47.64)^##^

Data are presented as means ± SD or medians (interquartile range). BMI, body mass index; TC, total cholesterol; LDL-C, low-density lipoprotein cholesterol; HDL-C, high-density lipoprotein cholesterol; TG, triglyceride; ApoA-Ⅰ, apolipoprotein A-I; ApoB, Apolipoprotein B; FBG, fasting blood glucose; FINS, fasting insulin; FCP, fasting C-peptide; HbA_1c_, Hemoglobin A1c; HOMA-IR, homeostasis model assessment of insulin resistance; HOMA-β, homeostasis model assessment of β-cell function.*P < 0.05 vs controls. **P < 0.01 vs controls. ^#^P < 0.05 vs baseline in the group with type 2 diabetes. ^##^P < 0.01 vs baseline in the group with type 2 diabetes.

### Lipidomic Analysis at Baseline

**Figure 1:**
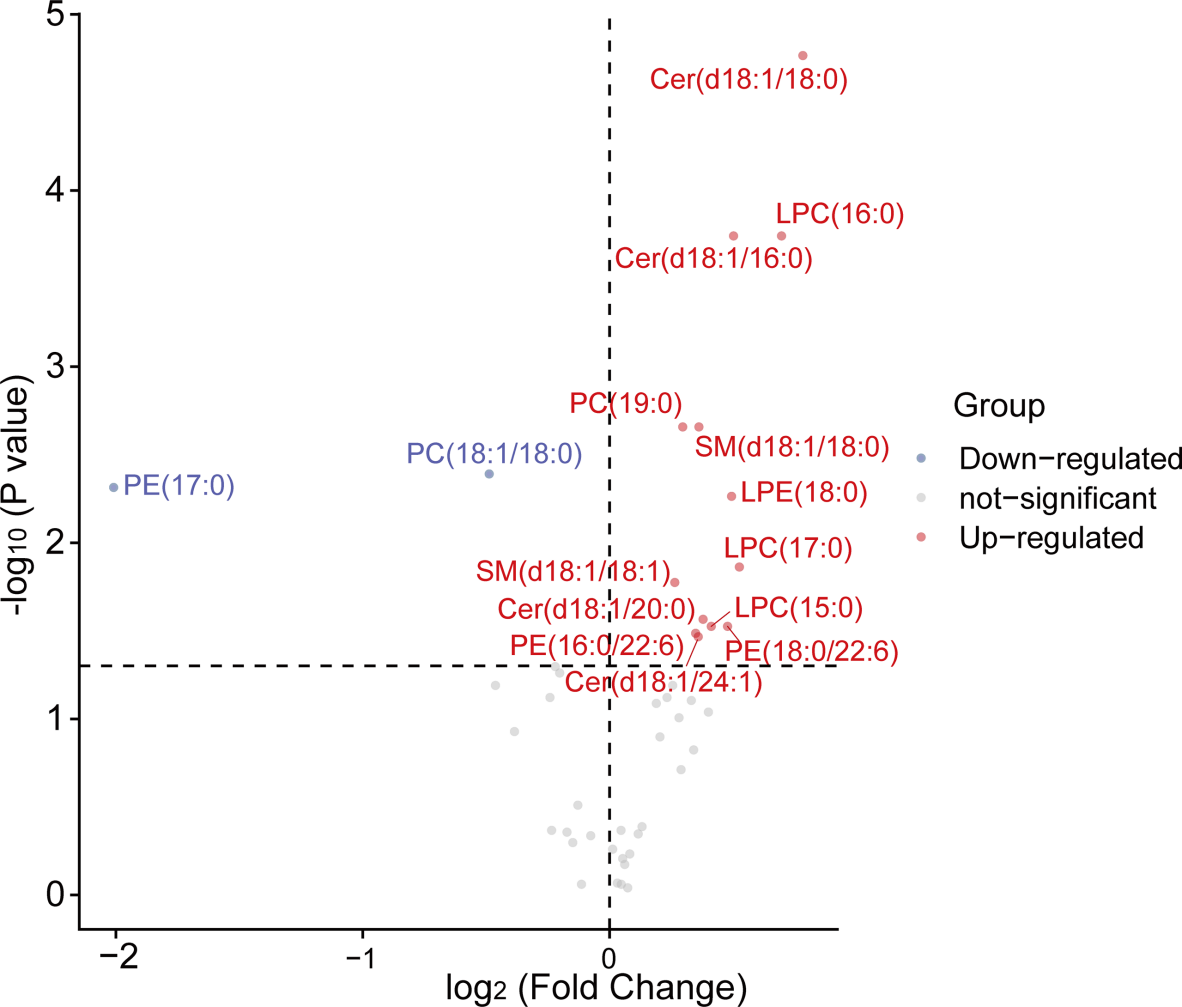
The volcano plots of 45 serum lipids levels between control and T2DM groups. A total of 15 lipids exhibited significant differential abundance (*P* < 0.05) with 2 of them down-regulated and 13 of them up-regulated when comparing T2DM patients to control. Mann-Whitney U tests were used to calculate statistical significance. Differentially lipid species with different fold changes were individually color-coded with up-regulated lipids in red, down-regulated in blue, and not-significant in grey. LPC, lysophosphatidylcholine; PE, phosphatidylethanolamine; LPE, lysophosphatidylethanolamine; PC, phosphatidylcholine; SM, sphingomyelin; Cer, ceramide.

A total of 45 lipid species including sphingomyelins (SMs), ceramides (CERs), lysophosphatidylcholines (LPCs), phosphatidylethanolamines (PEs), lysophosphatidylethanolamines (LPEs), phosphatidylcholines (PCs) were successfully identified and quantified in the serum lipidome of subjects. Compared to the healthy controls, 13 lipids [SM (d18:1/18:0, d18:1/18:1), Cer (d18:1/18:0, d18:1/16:0, d18:1/20:0, d18:1/24:1), LPC (15:0, 16:0, 17:0), PC (19:0/19:0), LPE (18:0) and PE (16:0/22:6, 18:0/22:6)] were markedly increased in the T2DM group, while PE (17:0/17:0) and PC (18:1/18:0) were decreased (*P* < 0.05, **Figure 1**).

### Correlations of T2DM-Related Differential Lipid Species and Clinical Characteristics

**Figure 2:**
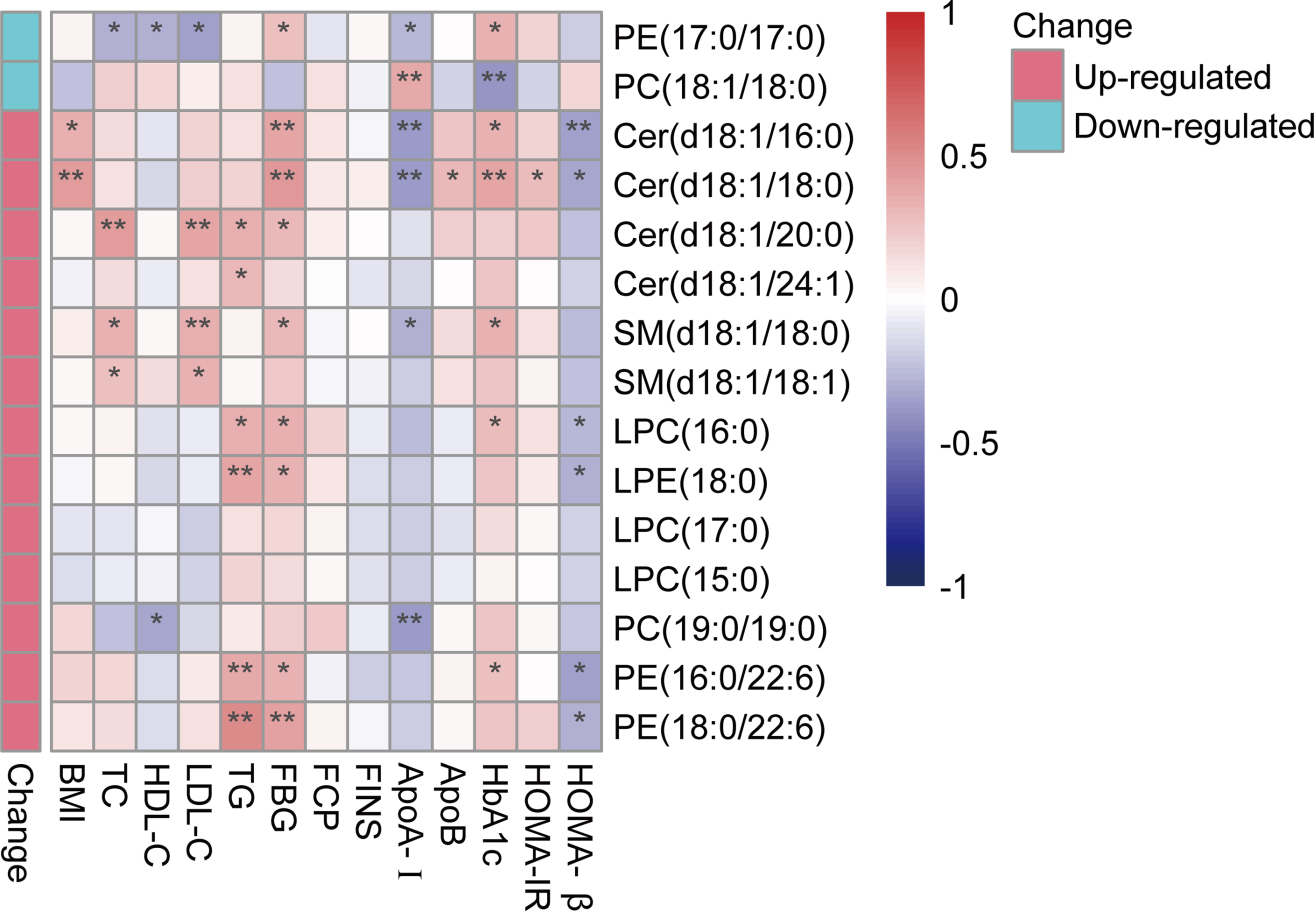
Correlation heatmap between differentially abundant lipids and key clinical characteristics. A bipolar color progression with scales on the right of the figure represented positive correlation (red) and negative correlation (blue), respectively. Statistical significances are indicated by asterisks. *P < 0.05; **P < 0.01. LPC, lysophosphatidylcholine; PE, phosphatidylethanolamine; LPE, lysophosphatidylethanolamine; PC, phosphatidylcholine; SM, sphingomyelin; Cer, ceramide.

Correlations of differential lipid species between T2DM and healthy controls with baseline metabolic parameters were investigated. Among them, nine of the differential lipid species from PE, CER, SM, LPC, and LPE were consistently positively correlated with FBG and six of up-regulated differential lipid species from PE, CER, LPC and LPE were consistently negatively correlated with HOMA-β. PE (17:0/17:0) and PC (19:0/19:0) were the only two species negatively correlated with parameters of lipid metabolism. ApoA-I was negatively correlated with four up-regulated species from PC, PE, CER, and SM while positively correlated with another species of PC (**Figure 2**).

### Effects of Exenatide on Clinical Parameters and Lipid Species on T2DM

**Figure 3:**
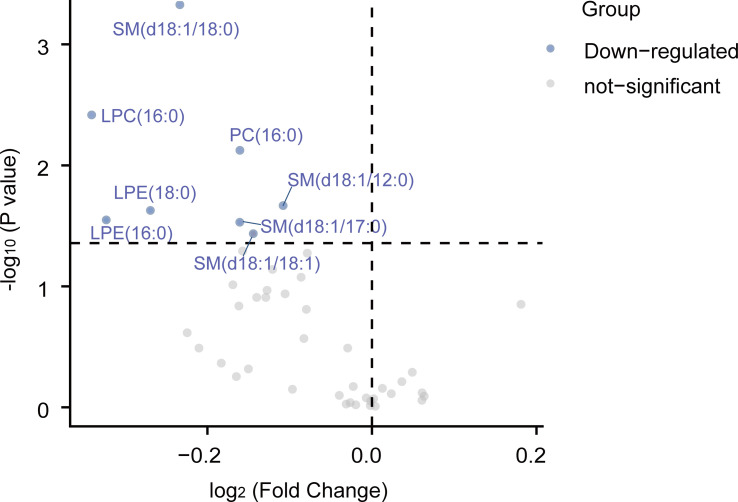
The volcano plots of 45 serum lipid levels inT2DM groups before and after treatment. A total of 8 lipids are down-regulated when comparing levels of post-treatment levels to pre-treatment levels. Paired Wilcoxon tests were used to calculate statistical significance. Differentially abundant metabolites with different fold changes were individually color-coded with down-regulated lipids in blue and not-significant in grey. LPC, lysophosphatidylcholine; PE, phosphatidylethanolamine; LPE, lysophosphatidylethanolamine; PC, phosphatidylcholine; SM, sphingomyelin; Cer, ceramide.

Changes in the clinical parameters after exenatide treatment in the T2DM group were shown in **Table 1**. BMI, TC, LDL-C, TG, ApoB, FBG, HbA_1c_, and HOMA-IR significantly decreased from baseline after 12 weeks of exenatide treatment, while HOMA-β increased (LDL-C, and ApoB: *P* < 0.05; BMI, TC, TG, FBG, HbA_1c_, HOMA-IR, and HOMA-β: *P* < 0.01, **Table 1**). From the volcano plot of **Figure 3**, exenatide treatment significantly improved serum levels of 8 lipid species [SM (d18:1/18:0, d18:1/18:1, d18:1/12:0, d18:1/17:0), LPC (16:0), LPE (18:0, 16:0), and PC (16:0/16:0)] in the T2DM group.

**Figure 4:**
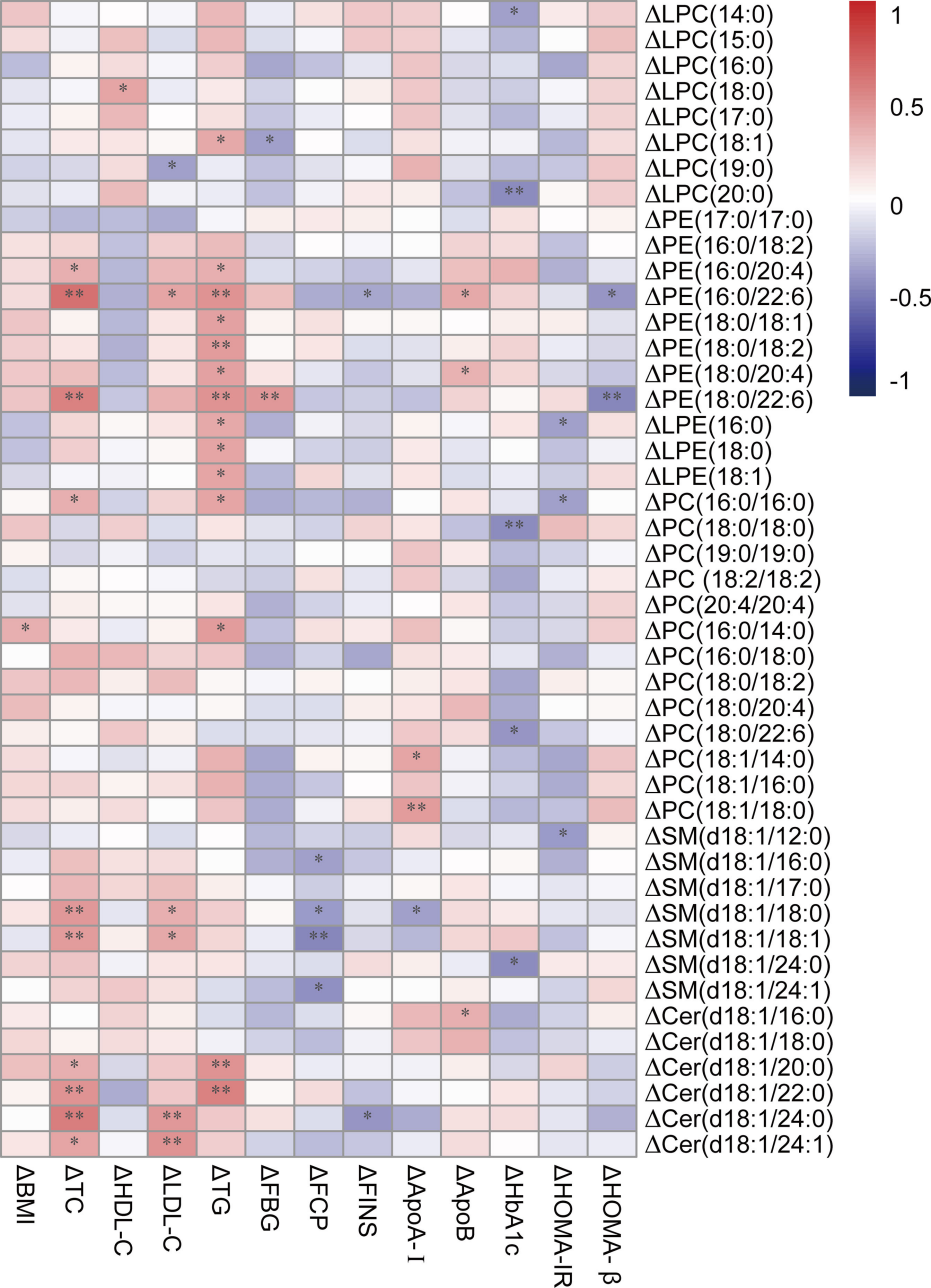
Correlation heatmap between differences of down-regulated lipids and key clinical characteristics. A bipolar color progression with scales on the right of the figure represented positive correlation (red) and negative correlation (blue), respectively. Statistical significances are indicated by asterisks. *P < 0.05; **P < 0.01. Δ means the differences of lipids or clinical parameters between before and after exenatide treatment. LPC, lysophosphatidylcholine; PE, phosphatidylethanolamine; LPE, lysophosphatidylethanolamine; PC, phosphatidylcholine; SM, sphingomyelin; Cer, ceramide.

The three down-regulated lipid species from SM, LPC, and LPE were significantly decreased after exenatide treatment, which was accompanied by the amelioration of lipids and glycemic parameters (TC, LDL-C, ApoA-I, FCP, and HbA_1c_) in T2DM patients (**Figure 4**).

### Pairwise Correlations Between Lipid Species

**Figure 5:**
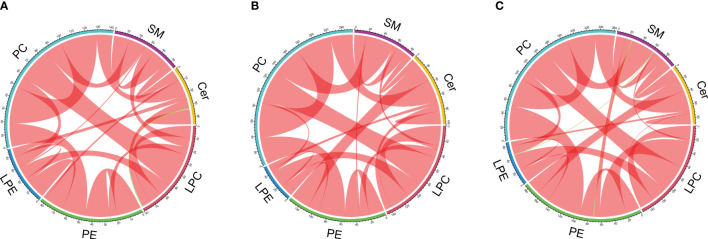
Chord diagrams of significant (P<0.05) pairwise correlations between lipids for the control group **(A)**, T2DM patients before exenatide treatment **(B)**, and T2DM patients after exenatide treatment **(C)** respectively. The colored arcs outside of the circle represent 6 lipid classes. Significant correlations between every pair of lipid species are depicted by sectors, with red representing positive correlation and green representing negative correlation. Bandwidth corresponds to the number of correlated pairs. LPC, lysophosphatidylcholine; PE, phosphatidylethanolamine; LPE, lysophosphatidylethanolamine; PC, phosphatidylcholine; SM, sphingomyelin; Cer, ceramide.

In addition, we analyzed the pairwise correlations between lipids in different groups and visualized their relationships using chord diagrams. In the chord diagrams, 6 lipid classes were arranged around the circle. The “hill-like” arc connections display the number of highly correlated lipids between two lipid classes, with different colors distinguishing positive and negative correlations. By generating chord plots, we can clearly observe the similarities and differences among the healthy controls (**Figure 5A**) and the before (**Figure 5B**) and after exenatide treatment groups (**Figure 5C**). Our results suggested that three groups presented similar lipid correlation patterns, in which most lipids are positive correlations with only a few negative correlations. However, the three groups also had substantial patterned differences, which might be related to the underlying pathway of lipid metabolism for T2DM and exenatide treatment. For instance, the positive correlations between PC and SM are stronger in healthy controls than in T2DM patients before treatment but restored after exenatide treatment. On the contrary, the positive correlations between PE and CER are stronger in T2DM patients before treatment than healthy controls but somewhat lost after exenatide treatment. Furthermore, negative correlations exist between LPC and CER only in the control group whereas between PE and SM only in the T2DM group after treatment. Therefore, lipidomic correlation patterns provided more comprehensive molecular insights into assessing the efficacy of exenatide.

## Discussion

In the current study, we observed that serum lipidomic profiles were substantially altered in T2DM patients compared with the healthy controls. The differential lipid species were correlated with improvement of glycolipid metabolism and insulin resistance/sensitivity indicators. Exenatide treatment for 12 weeks decreased 4 serum levels of lipid species from SM, LPC, and LPE, which were significantly increased in T2DM patients. Our findings provide new insight into a broad range of lipid molecules into the potential candidate mechanisms driving the benefits of exenatide treatment.

Given the established link between T2DM and dysregulation of lipid metabolism (4), it is not surprising that many studies have identified strong relationships between circulating lipids and T2DM with lipidomic analysis (24, 25). The six main categories of tested lipids were SMs, CERs, LPCs, PEs, LPEs, and PCs. Each of these categories contains many individual molecular species. Compared to the healthy controls, 13 lipid species of SM, CER, LPC, PC, LPE, and PE were markedly increased in the T2DM group, while two lipid species from PE and PC were decreased, most of which were correlated with parameters of glycolipid metabolism and insulin resistance/sensitivity indicators.

Ceramides are precursors of complex sphingolipids and are considered to be involved in insulin resistance and lipotoxicity (26). A large cohort study of 2302 participants with or without diabetes demonstrated that ceramides positively correlated with BMI and HOMA-IR in patients with T2DM (27). Moreover, Yun H et al. examined the plasma levels of 76 sphingolipids in 1,974 participants and showed that elevated plasma CERs and SMs levels are largely mediated through β-cell dysfunction in diabetic patients (10). In line with the prior studies, our study showed that high levels of ceramides correlated with abnormal glucolipid metabolism and insulin resistance in T2DM patients. The mechanism mainly involves Ceramide Synthases through their regulation of FAO and insulin signaling in the context of T2DM (28–30). Interestingly, we also found that two species of ceramides positively correlated with ApoA-I, but not correlated with HDL. ApoA-I, a major protein of HDL, and plays a critical role in improving cellular cholesterol efflux along with atheroprotective functions. Clinical studies reported that circulating ceramides may be a novel biomarker of cardiovascular diseases (31, 32). These results implied that specific ceramides may increase the risk of cardiovascular outcomes in T2DM. Further studies are needed to confirm this possibility.

In another large plasma lipidomic profiling study consisting of healthy controls, prediabetes, and T2DM patients, a total of 259 lipid species from 1,427 human plasma samples were profiled (33). It is reported that Ceramides were positively associated with T2DM, and lipid species of PE were also identified to be increased in T2DM and had positive correlations with FBG. PEs along with PCs and SMs are major components of the cell membrane lipids. Dysfunction of PE and PC have been found in obesity (34) and non-alcoholic fatty liver disease (35), which are usually linked to dyslipidemia and T2DM (33). A case-cohort study nested within the PREDIMED trial, with 250 incident T2DM cases diagnosed and a random sample of 692 participants showed that PEs and LPEs were positively associated with T2DM risk (36). We observed that exenatide decreased serum LPE (18:0) in T2DM patients, which was positively related to TG. Singh AB et al. showed Liver-specific knockdown ACSL4 altered liver LPC species (LPC 16:0 and LPC 18:0) and LPE species (LPE 16:0 and LPE 18:0), and decrease in circulating VLDL-TG levels in high-fat diet mice (37). The *db/db* mice were treated with Kukoamine B showed reduced PE with increased PC and had reduced systemic inflammation through regulating nuclear transcription factors (38). All the above results suggested that there are lipidomic perturbations in patients with T2DM.

Exenatide was the first commercialized drug developed as the GLP-1 mimetics. Clinical evidence substantiating exenatide is an efficacious agent to treat T2DM with abnormal lipid metabolism (39, 40). Gonza´lez-Ortiz et al. have reported one-month administration of exenatide significantly reduced TC and LDL-C in patients with metabolic syndrome (41). Moreover, obese T2DM patients who received exenatide therapy for 6 months in another study also showed dramatically ameliorated glucose and lipid metabolism, improved insulin sensitivity (42). In addition, exenatide treatment for more than 3 years in patients with T2DM leads to continuous improvements in lipid profile, coupled with progressive reduction of weight (43). All of these studies indicated that both short-term and long-term exenatide treatment improved lipid metabolism. In accordance with the abovementioned findings, our results showed that elevated serum levels of SM (d18:1/18:0, d18:1/18:1), LPC (16:0), and LPE (18:0) in T2DM patients, were significantly decreased after 12-week exenatide treatment. The decreases we observed in several SM, LPC, and LPE species correlated with the improvement in insulin and glucose metabolism indicators, suggesting that these lipid species may contribute to the pathogenesis of T2DM and may serve as a novel promising therapeutic biomarker for T2DM.

SMs, which can be hydrolyzed to produce ceramides, were significantly decreased in serum after exenatide treatment. SMs have been reported to induce cell dysfunction through altered cellular signaling, promotion of proliferation, migration, inflammation, and cell survival, all of which could adversely affect organ function (44, 45). Consistent with our study, Somm E et al. reported another GLP-1R agonist liraglutide limits the accumulation of ceramides/sphingomyelins species in the liver of MCD diet-fed mice (46). Another double-blind placebo-controlled trial of 102 participants with T2DM reported that liraglutide downregulates circulating ceramides, phospholipids, and triglycerides, which further strengthens the recommendation of GLP-1 receptor agonists to prevent cardiovascular disease in T2DM (47). Moreover, we also observed a decreased trend of ceramides albeit without significance, which indicated that exenatide may inhibit SMs biosynthesis and Ceramides synthesis, further assisting in improving dyslipidemia. However, the detailed mechanisms require further investigation.

Lipid species related to phospholipid metabolism are important parts of the lipidome that was involved in the progression and therapy of T2DM (48, 49). Prior to treatment, we found that the T2DM group had higher levels of LPC (15:0, 16:0, 17:0) and LPE (18:0) than the control group. In contrast, the T2DM group after exenatide treatment was associated with lower levels of LPC (16:0) and LPE (18:0), indicating a shift in phospholipid metabolism after exenatide treatment. Recently, Razquin C et al. found that the changes of plasma LPC and LPE were related to the risk of T2DM (36). Lysophospholipids (namely LPC, LPE), is important for glucose-mediated insulin secretion in diabetes targeting tissues (48). Therefore, higher levels of these two lysophospholipids may induce β-cell failure, metabolic inflexibility, and thereby induce insulin resistance and islet dysfunction (50). Our finding of changed lysophospholipids before and after exenatide treatment could be a marker of both changed glucose-mediated insulin secretion and metabolic flexibility in T2DM.

In addition, our quantitative lipidomic information enables in-depth profiling of lipids, showing significant exenatide-specific lipid patterns. The chord diagrams demonstrated that parallel rather than individual lipidomic profiling provides more comprehensive molecular insights into assessing the efficacy of exenatide. We believe that the changes of lipid correlation patterns in this study allow circumventing some lipid classes or subclasses. Our findings strengthen lipidomics as a promising tool for exploring molecular mechanisms underlying T2DM. It could be applied to other T2DM lipidomics research to better understand lipid metabolic patterns and integration with other omics measurements.

The current study has some limitations. First, it is relatively small, which may due to the absence of differences in other lipid species. Second, the short duration of follow-up could affect the durable and potential role of lipidomics as a therapeutic tool; hence a longer clinical study is needed. Third, all participants in this study were Chinese, so further work is needed to determine whether these findings could be extrapolated to other ethnicities. Therefore, further larger clinical and basic research are required to elucidate the mechanisms underlying exenatide and other GLP-1 receptor agonists (liraglutide, dulaglutide, semaglutide) on specific lipids.

## Conclusion

In summary, our study confirmed that T2DM patients with obesity have broadly lipidomic perturbations. Exenatide improved lipid metabolism, especially SMs, LPCs, and LPEs, all of which are potential lipid species that may provide novel insight to elucidate the beneficial effects of exenatide.

## Data Availability Statement

The raw data supporting the conclusions of this article will be made available by the authors, without undue reservation.

## Ethics Statement

The studies involving human participants were reviewed and approved by Ethics Committee of Beijing Chao-yang Hospital Affiliated with Capital Medical University. The patients/participants provided their written informed consent to participate in this study.

## Author Contributions

GW and JL conceived and designed the experiments. LZ and YH performed the experiments. LZ, YA, and QW analyzed and interpreted the patient data. LZ wrote the manuscript. All authors read and approved the final manuscript.

## Funding

This work was supported by grants from the Chinese National Natural Science Foundation (No. 81770792) and Key Projects of Science and Technology Planning of Beijing Municipal Education Commission (KZ201810025038) to GW; and the Beijing Talents foundation (2018-12) to JL.

## Conflict of Interest

The authors declare that the research was conducted in the absence of any commercial or financial relationships that could be construed as a potential conflict of interest.

## Publisher’s Note

All claims expressed in this article are solely those of the authors and do not necessarily represent those of their affiliated organizations, or those of the publisher, the editors and the reviewers. Any product that may be evaluated in this article, or claim that may be made by its manufacturer, is not guaranteed or endorsed by the publisher.

## Abbreviations

T2DM, type 2 diabetes mellitus; GLP-1, glucagon-like peptide-1; UPLC-QTOF-MS, ultrahigh-performance liquid chromatography equipped with quadrupole time-of-flight mass spectrometry; BMI, body mass index; HbA1c, glycohemoglobin; QC, Quality control; CV, coefficient of variation; TC, total cholesterol; LDL-C, low-density lipoprotein cholesterol; HDL-C, high-density lipoprotein cholesterol; TG, triglyceride; ApoA-I, apolipoprotein A-I; ApoB, Apolipoprotein B; FBG, fasting blood glucose; FINS, fasting insulin; FCP, fasting C-peptide; HOMA-IR, homeostasis model assessment of insulin resistance; HOMA-β, homeostasis model assessment of β-cell function; LPC, lysophosphatidylcholine; PE, phosphatidylethanolamine; LPE, lysophosphatidylethanolamine; PC, phosphatidylcholine; SM, sphingomyelin; CER/Cer, ceramide.
