# Supplementary material for: The Changes of Lipidomic Profiles Reveal Therapeutic Effects of Exenatide in Patients With Type 2 Diabetes
Source: Front Endocrinol (Lausanne). 2022 Mar 31;13:677202. doi: 10.3389/fendo.2022.677202 (PMC9009038; doi:10.3389/fendo.2022.677202)
Supplement: Supplementary file 1 [file DataSheet_1.doc]

***Supplementary Material***

**Lipidomics profiling**

***Sample Preparation.*** Lipids were extracted from 20 µl serum by 5-fold cold chloroform: methanol (2:1) solution containing 1 µg/mL LPC (19:0), PC (19:0/19:0), PE (17:0/17:0) and SM (d18:1/12:0) (Avanti Polar Lipids, Alabaster, AL) as internal standards. The samples were vortexed for 30 sec and then allowed to stand for 30 min at 4℃. The mixture was centrifuged at 7,800 g/min for 3 min and then the lower organic phase was collected and evaporated at room temperature under vacuum and the residue was dissolved in chloroform: methanol (1:1), followed by diluting with isopropanol: acetonitrile (2:1) to lipidomic analysis. The clinical samples were injected into the analytic workflow randomly. Quality control (QC) sample was injected between every 15 samples to assure that the MS signal for evaluating the reliability of the method, features with CVs over 15% was eliminated.

***UPLC-QTOF-MS Analysis*.** UPLC-QTOF-MS analysis was performed using an LC-20AXR Rapid Separation LC system (Shimadzu, Kyoto, Japan) coupled with an AB Triple quadrupole time-of-flight 5,600 mass spectrometer (AB SCIEX, Foster City, CA, USA). Chromatographic separation was performed using a Waters XBridge BEH C18 (3.5μm, 2.1×100mm) under the following conditions: UPLC: A, water (100); B, isopropanol/acetonitrile (50:50); both A and B contained 10 mM ammonium acetate and 0.1% formic acid. Gradient: initial 65% A to 20% A at 2 minutes, to 0% A at 9 minutes*, to 0% A at 15 minutes*, to 65% A at 16 minutes with equilibration for 4 additional minutes (asterisk indicates the ballistic gradient). The flow rate was 0.4 ml/minute. The column temperature was maintained at 55°C. An electrospray ionization source (ESI) operating in negative ion modes was used in MS analysis. The ion source parameters were Curtain Gas = 30 psi, Ion Source Gas 1 = 50 psi, Ion Source Gas 2 = 50 psi, Ion Spray Voltage = −4500 V, and Source Temperature = 550 °C. The data were collected with a range of 100 to 1,500 *m/z*.

***Data quantification.*** The acquired mass data were processed by Analysis software (SCIEX, USA) for peak detection, calibration, and normalization. After excluding those lipids with coefficients of variations (CV) > 15%, we profiled 45 lipid species including sphingomyelins (SMs), ceramides (CERs), lysophosphatidylcholines (LPCs), phosphatidylethanolamines (PEs), lysophosphatidylethanolamines (LPEs), phosphatidylcholines (PCs).
